# utPCR: A Strategy for the Highly Specific and Absolutely Quantitative Detection of Single Molecules within Only Minutes

**DOI:** 10.3390/bios13100910

**Published:** 2023-09-27

**Authors:** Rui Wang, Ying Liu, Shuaiwei Chen, Linlin Bai, Kaiming Guo, Yanan Pang, Feng Qian, Yongfang Li, Li Ding, Yongming Wang

**Affiliations:** 1 State Key Laboratory of Genetic Engineering, School of Life Sciences, Zhongshan Hospital, Human Phenome Institute, Pudong Hospital, Fudan University, Shanghai 200438, China; wangr@fudan.edu.cn (R.W.); liuyinglyac@163.com (Y.L.); 21210700106@m.fudan.edu.cn (S.C.); 18437953528@163.com (L.B.); 18307110089@fudan.edu.cn (K.G.); fengqian@fudan.edu.cn (F.Q.); 2 School of Engineering and Applied Sciences, Harvard University, Cambridge, MA 02138, USA; 3 Changhai Hospital, Second Military Medical University, Shanghai 200433, China; 15721259472@163.com; 4 School of Food Science and Engineering, Foshan University, Foshan 528231, China; 18110220111@fudan.edu.cn; 5 Shanghai Engineering Research Center of Industrial Microorganisms, Shanghai 200438, China

**Keywords:** ultrafast PCR, droplet digital PCR (ddPCR), single-molecule detection, bloodstream infection, absolute quantification, molecular diagnosis

## Abstract

Bloodstream infection is a major health problem worldwide, with extremely high mortality. Detecting infection in the early stage is challenging due to the extremely low concentration of bacteria in the blood. Digital PCR provides unparalleled sensitivity and can achieve absolute quantification, but it is time-consuming. Moreover, the presence of unavoidable background signals in negative controls poses a significant challenge for single-molecule detection. Here, we propose a novel strategy called “Ultrafast flexible thin tube-based droplet digital PCR (utPCR)” that can shorten the digital PCR process from 2 h to only 5 min, with primer annealing/extension time reduced from minutes to only 5 s. Importantly, the ultrafast PCR eliminates nonspecific amplification and thus enables single-molecule detection. The utPCR enabled the sensitive detection and digital quantification of *E. coli* O157 in the high background of a 10^6^-fold excess of *E. coli* K12 cells. Moreover, this method also displayed the potential to detect rare pathogens in blood samples, and the limit of detection (LOD) could be as low as 10 CFU per mL of blood without false positive results. Considered ultrafast (<5 min) and highly sensitive (single-molecule detection), the utPCR holds excellent prospects in the next generation of molecular diagnosis.

## 1. Introduction

Bloodstream infection is a significant global health issue, with a mortality rate of up to 20% [1,2,3,4]. The rapid detection of bacteria, especially in the early stages of infection, is crucial. However, the traditional culture method is complex and time-consuming, taking several days to yield results, thus making it unsuitable for prompt diagnosis [5,6,7,8]. Recently, amplification-based molecular diagnostic methods, such as polymerase chain reaction (PCR), have been developed to reduce the assay time from days to hours [9,10,11,12,13,14]. Nonetheless, conventional PCR lacks the sensitivity required to detect bacteria present in low concentrations in the bloodstream (1~100 CFU per mL). Additionally, it tends to preferentially amplify short fragments and generate short chimeric molecules, which pose a significant challenge for single-molecule analysis when the template concentration is low [15].

Presently, digital PCR has emerged as a novel nucleic acid quantitative technique [16,17,18]. In digital PCR, DNA templates are randomly distributed into millions of small chambers or monodisperse droplets prior to amplification [14,19,20]. Theoretically, reaction compartments containing template molecules will exhibit a fluorescence signal after amplification, while those without template molecules will remain dark. Digital PCR offers unparalleled sensitivity and enables absolute quantification detection. However, most digital PCR processes still require several hours to complete [21,22]. Furthermore, in practical applications, there is typically some inevitable fluorescence present in negative controls during digital PCR detection, significantly interfering with result interpretation, particularly for trace detection [23,24,25]. Consequently, there is an urgent need for a faster, more specific, and extremely sensitive strategy for single-molecule amplification and analysis in the presence of a high background substrate.

**Scheme 1:**
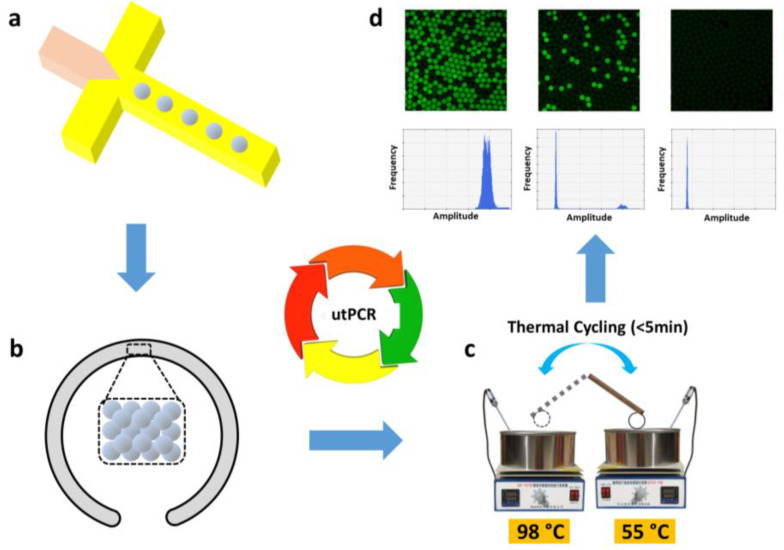
Schematic of ultrafast and accurate quantitation of rare target from the high background with the proposed utPCR method. (**a**) Targets with background DNA were randomly distributed into millions of monodisperse droplets with the help of PDMS device. (**b**) All droplets were transferred into the flexible thin tube. (**c**) The flexible tube was shuttled between two water baths to make ultrafast PCR amplification. (**d**) After reaction, the droplets were observed under confocal or read by droplet reader.

In this study, we have developed a novel strategy called “Ultrafast flexible thin tube-based droplet digital PCR (utPCR)”. As illustrated in Scheme 1, the detection process involved the random distribution of targets with background DNA into millions of monodisperse droplets. These droplets were then transferred into a flexible thin tube to facilitate ultrafast PCR thermal cycling. Following the reaction, the droplets were either observed under a confocal microscope or read by a droplet reader. Due to the high heat conduction efficiency of the flexible thin tube, ultrafast PCR could be completed within 5 min. The reduction of PCR annealing time from minutes to seconds helped to mitigate nonspecific amplification to some extent. In negative samples, all droplets remained nonfluorescent, thus enabling single-molecule detection.

## 2. Materials and Methods

### 2.1. Reagents and Chemicals

Strains of *E. coli* O157 (ATCC 700728) and *E. coli* K12 (ATCC 700926) were generously provided by Harvard University (Cambridge, MA, USA). Fresh whole blood was obtained from Research Blood Components, LLC. (Watertown, MA, USA).

The nucleic acid extraction kit was obtained from QIAGEN Inc. (Germantown, MD, USA). For DNA amplification and analysis, all primers were synthesized by Integrated DNA Technologies, Inc. (Skokie, IL, USA). TaKaRa SpeedSTAR™ polymerase was purchased from Takara Bio Inc. (San Jose, CA, USA). The EvaGreen^®^ dye was obtained from Biotium, Inc. (Fremont, CA, USA). The UltraPure™ agarose was provided by Thermo Fisher Scientific Inc. (Waltham, MA, USA). The GenElute™ gel extraction reagent was bought from Sigma-Aldrich, Inc. (St. Louis, MO, USA). The DNA amplicons were sequenced by GENEWIZ LLC. (South Plainfield, NJ, USA). Bovine serum albumin (BSA) was bought from Thermo Fisher Scientific Inc. (MA, USA). Tween 20 was purchased from Sigma-Aldrich, Inc. (MO, USA). The surfactant was purchased from RAN Biotechnologies, Inc. (Beverly, MA, USA). HFE-7500 3M^TM^ Novec^TM^ engineered fluid was provided by 3M Corp. (St. Paul, MN, USA). The carrier oil used for droplet generation, HFE-7500, contained 5% (*w*/*w*) surfactant.

For microfluidic device fabrication, silicon wafers were supported by University Wafer, Inc. (South Boston, MA, USA). SU-8 reagents were provided by MicroChem Corp. (Newton, MA, USA). Isopropyl alcohol and BAKER BTS-220 were obtained from Avantor Performance Materials, LLC. (Allentown, PA, USA). Sylgard 184 Silicone Encapsulant Clear was provided by Dow Corning Corp. (Midland, MI, USA).

Bacterial culture was performed with Bacto™ Tryptone, Bacto™ yeast extract and Difco™ agar that was obtained from Becton, Dickinson, and Company (Sparks Glencoe, MD, USA). Phosphate-buffered saline (PBS) was purchased from Corning Inc. (New York, NY, USA), and sodium chloride (NaCl) was obtained from Sigma-Aldrich, Inc. (MO, USA).

For digital PCR amplification, a flexible thin tube was purchased from Scientific Commodities, Inc. (Lake Havasu City, AZ, USA). For the tube, the inner diameter is 0.4 mm, the wall thickness is 0.15 mm, the material is polypropylene, and the thermal conductivity is 0.21 Wm^−1^K^−1^. All water baths and thermo blocks were obtained from Thermo Fisher Scientific Inc. (MA, USA). The CFX96 fluorescent system, QX200™ droplet reader, and ChemiDoc XRS+ System were purchased from Bio-Rad Laboratories, Inc. (Hercules, CA, USA). The NanoDrop ND-1000 was obtained from Thermo Fisher Scientific Inc. (MA, USA). The Leica TCS SP5 confocal microscope was purchased from Leica Microsystems Inc. (Buffalo Grove, IL, USA). Syringe pumps were provided by KD Scientific Inc. (Holliston, MA, USA).

For device fabrication, the 2105C2 Illumination Controller was obtained from Radiation Power Systems, Inc. (San Jose, CA, USA), and the Plasma Prep2 was purchased from Diener Electronic GmbH +Co. KG (Ebhausen, Germany).

### 2.2. Design and Fabrication of the Microfluidic Device and Droplet Generation

The PDMS microfluidic devices were fabricated using soft lithographic techniques. The design of the microfluidic channels in PDMS was created using AutoCAD. In preparation, the channel architectures were transferred onto high-resolution photomasks. Different photoresist SU-8 reagents were spin-coated onto silicon wafers, taking into account the desired thickness for each channel. After a soft bake, the silicon wafers were patterned through UV exposure using the photomask. The patterned silicon wafers were then baked at 65 °C, followed by 95 °C. Subsequently, the silicon wafers underwent development by immersion in BAKER BTS-220. Once sufficiently washed and dried, the silicon wafers were placed in Petri dishes and filled with a mixture of pre-polymer and cross-linker (*w*/*w*, 10:1). After degassing in a vacuum desiccator chamber, the Petri dishes were heated at 65 °C for 2 h to form structured microfluidic substrates. The resulting PDMS casts were then peeled off and punched to create inlet and outlet ports. The channel side of the PDMS cast was subsequently attached to a clean glass slide through plasma bonding. The specific details of the reaction chambers can be found in Table 1, which indicate different channel lengths and widths. Following bonding, the microfluidic channels were treated with aquapel to render them hydrophobic and dried using nitrogen gas.

**Figure 1:**
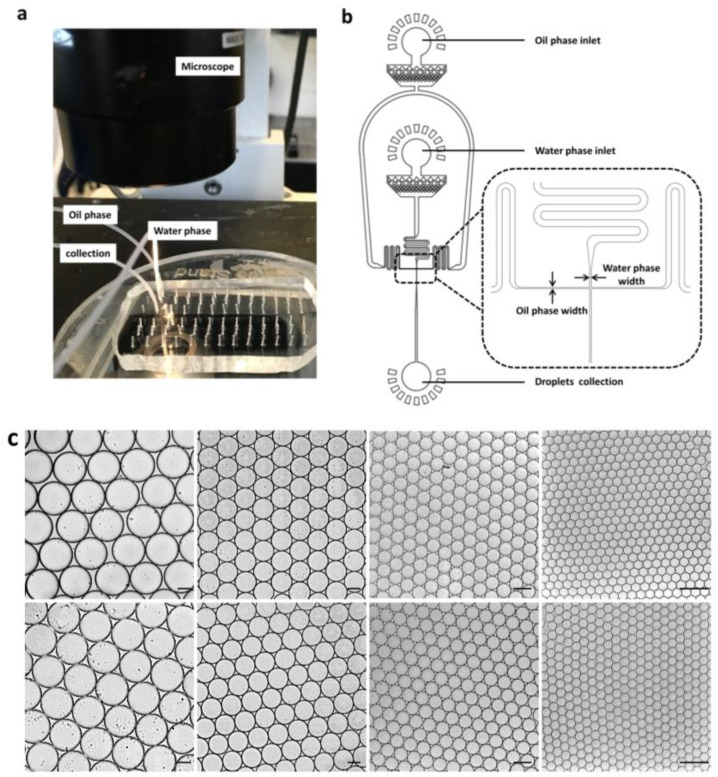
The actual image (**a**) and cross-sectional schematic view (**b**) of a PDMS microfluidic device for monodisperse droplets generation. (**c**) Microscope images of droplets before (top) and after (bottom) PCR thermal cycling. From left to right, the diameter of droplets were 120 μm, 80 μm, 37 μm, and 15 μm, respectively. Scale bar: 50 μm.

For the generation of droplets, the oil phase and water phase were introduced into a PDMS device through their respective inlets at constant flow rates. Upon reaching the intersection, the oil phase effectively segmented the water phase into individual droplets. Subsequently, all the formed droplets and remaining liquid were collected at the outlet (Figure 1a,b).

### 2.3. Bacterial Culture and Spiked Sample Preparation

A Luria-Bertani (LB) medium was prepared prior to the bacterial culture. The liquid LB medium was composed of 1% (*w*/*v*) Tryptone, 1% (*w*/*v*) NaCl, and 0.5% (*w*/*v*) yeast extract. The LB agar plates additionally contained 1.5% (*w*/*v*) agar. All LB media were sterilized at 121 °C for 20 min, then placed at 4 °C before use.

Two strains of bacteria, namely *E. coli* O157 and *E. coli* K12, were cultured individually in 5 mL of liquid LB medium overnight at 37 °C. Subsequently, the cultured bacteria were separately collected after being washed three times with sterilized PBS, then centrifuged at 5000 rpm for 20 min. The bacterial pellets were placed in 5 mL PBS and gradually diluted ten-fold for subsequent spiking assays.

The concentration of bacteria in each dilution was determined in triplicate by measuring the UV absorption at 600 nm. In parallel, 50 μL of each bacterial dilution was spread on LB plates for colony counting after overnight incubation. The bacterial concentration measured via UV absorption was further calibrated using the bacterial colony counting method.

For rare pathogen detection in the presence of high background interference, *E. coli* O157 and *E. coli* K12 were uniformly mixed, with *E. coli* O157 concentration ranging from 5 × 10^4^ to 5 CFU/mL, while *E. coli* K12 was maintained at a concentration of 5 × 10^6^ CFU/mL. Each mixture was divided into six aliquots. Following DNA extraction, three aliquots from each mixture were analyzed using utPCR, while the remaining aliquots were subjected to standard droplet PCR as a control.

In the spiking assay using blood samples, 100 μL of serial 10-fold dilutions of *E. coli* O157 suspension were mixed with 900 μL of whole blood samples, respectively. Each spiking concentration was performed in six replicates and thoroughly mixed. After DNA extraction, three samples from each spiking concentration were analyzed using utPCR, while the remaining three samples were tested using standard droplet PCR as a control.

### 2.4. Nucleic Acid Extraction

Before nucleic acid extraction, all samples were centrifuged at 12,000 rpm for 2 min with supernatant discarded. Then, nucleic acid was extracted with a QIAamp^®^ DNA Mini Kit. DNA templates were dissolved in sterile water and adjusted to a final volume of 20 μL. For each PCR assay, 10 μL of DNA was used as the template, and the total PCR reaction volume was 25 μL.

### 2.5. The utPCR and Standard PCR Amplification

The forward primer (5′-TGGCAGGAAGAGAGTGACAGG-3′) and reverse primer (5′-GGCCTTACCCGTGAACAGTA-3′) targeting the *E. coli* O157 gene were designed using Primer Premier 5.0. The reaction conditions for utPCR and standard droplet PCR are compared in Table S1. To achieve ultrafast nucleic acid amplification, the concentration of DNA polymerase in utPCR was ten times that of standard PCR [10,26]. It was performed by hot start at 98 °C for 30 s, then carried out for 40 cycles of denaturation (98 °C for 1 s/2 s/5 s/30 s) and annealing/extension (55 °C for 2 s/3 s/5 s/30 s), respectively. To facilitate ultrafast PCR operation, the flexible thin tube was attached to the top of a stick and manually moved back and forth rapidly between two water baths set at different temperatures. Following the reaction, the droplets were transferred onto glass slides for observation using a confocal microscope or directly read by a droplet reader. The standard droplet PCR was carried out with hot start at 95 °C for 3 min, then 40 cycles including denaturation at 95 °C for 30 s, annealing at 55 °C for 30 s and extension at 72 °C for 1 min, with a final extension at 72 °C for 5 min.

### 2.6. Detection of Blood Stream Infection with the Proposed utPCR Method

We spiked blood samples with 10-fold gradient diluted *E. coli* O157 from 10^6^ to 1 CFU per milliliter of blood, with no *E. coli* O157 spiked blood sample as the negative control. DNA templates from each sample were extracted with a Qiagen mini-DNA extraction kit and suspended in 20 μL of sterile water. For each utPCR reaction, 10 μL of DNA was added as a template, and the total reaction volume was 25 μL. Each sample was tested by dividing the template into two aliquots for utPCR amplification, and then the copy numbers were added together. To match the requirement of fluorescence flow cytometry analysis by the commercial QX200TM Droplet Reader, the droplets generated in this assay were 120 μm in diameter. The utPCR amplification was carried out in a flexible tube within 5 min with standard droplet PCR amplification (~2 h) as control. After amplification, all droplets were read individually by the QX200TM Droplet Reader. The copy number of each reaction was calculated by the number and fraction of positive droplets according to Poisson distribution.

### 2.7. Data Acquisition and Analysis

After utPCR and standard droplet PCR amplification, the droplets were observed on a confocal platform. Fluorescent images were captured using a 10× objective under 470 nm fluorescence excitation. All images were analyzed using Image J and MATLAB. Droplets with a diameter of 120 μm underwent direct fluorescence flow cytometry analysis using the commercial QX200™ Droplet Reader. The concentration of template was calculated using the Poisson equation: P (X = k) = e^−λ^λ^k^(k!)^−1^, and N = −ln(1−a/b)b, where λ represents the average pathogen concentration in each droplet, a and b are the numbers of positive and total droplets in the sample, and N is the absolute number of pathogens in the sample.

## 3. Results

### 3.1. Monodisperse Droplets Generation and Stability Evaluation

First of all, to evaluate the stability of the droplets in the flexible thin tube during utPCR amplification, four types of droplets with diameters of 120 μm, 80 μm, 37 μm, and 15 μm, respectively, were employed to simulate the thermocycling process. All droplets were generated with PDMS devices with different diameters. Most of the droplet generation times were less than 5 min (Table 1, video). The video presented a demonstration of the swift generation of monodisperse droplets. The generated droplets were uniform under a microscope before thermal cycling (Figure 1). Then, they were transferred into the flexible thin tube and thermal cycling by hot start was carried out at 98 °C for 30 s, and then 40 cycles of 98 °C for 30 s and 55 °C for 30 s were carried out. The droplets were observed and photographed under a microscope after the thermal cycling process. The results showed that after thermocycling, all types of droplets were still uniform, demonstrating strong tolerance to the shuttling process. The droplet sizes were almost the same before and after PCR thermal cycling. We assumed that the surfactant in the carrier oil strongly prevented droplet merging and the ultrafast thermal cycling process greatly lowered the rate of droplet expansion. Therefore, the results verified that it is possible to achieve utPCR with the help of the flexible thin tube. Considering droplet generation time and detection accuracy, we chose droplets with 37 μm diameter for the following assay.

### 3.2. Ultrafast Droplet PCR Build-Up

**Figure 2:**
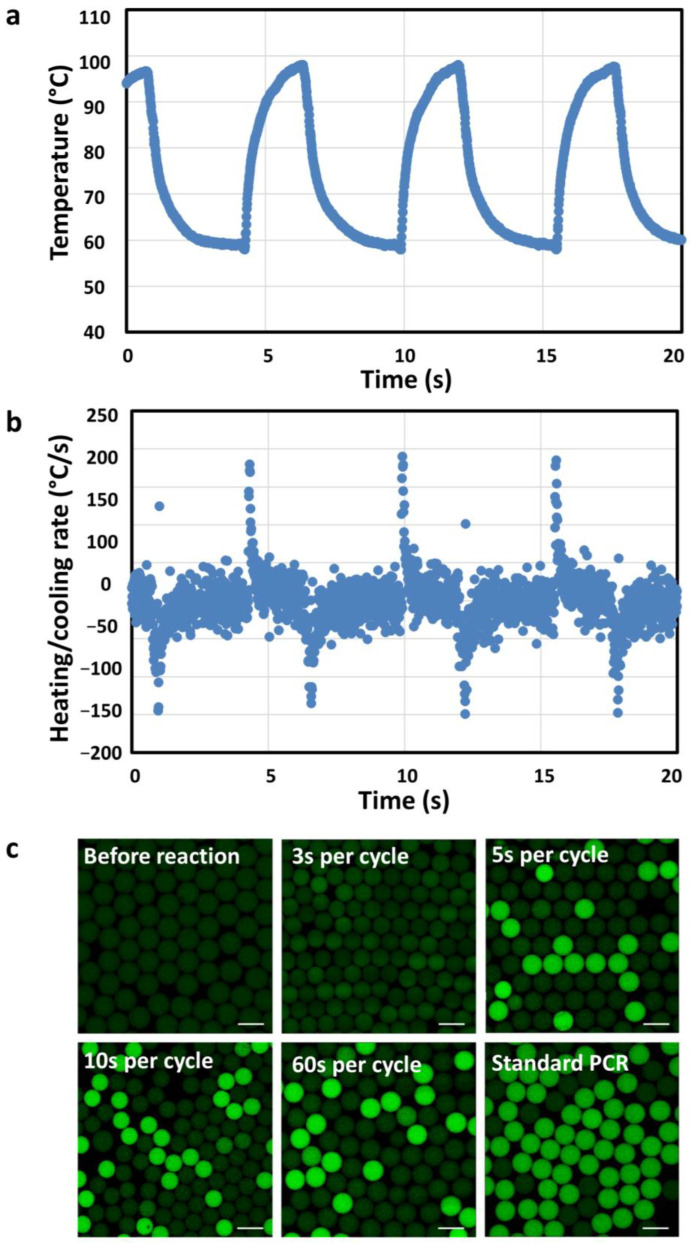
(**a**) The real-time temperature (**a**) and heating/cooling rate (**b**) in flexible thin tube with PCR thermal cycling process of 98 °C for 2 s and 55 °C for 3 s. (**c**) Confocal images of droplets before and after utPCR amplification with standard droplet PCR as control (scale bar: 50 μm).

Then, we explored the optimal thermal cycling time for utPCR reaction in the flexible thin tube. The details of the reaction conditions can be seen in Table S1. We tracked the temperature in the flexible thin tube during PCR thermal cycling with thermocouple. The result indicated that the maximum heating/cooling ramp rates inside the flexible thin tube could be above 150 °C/s (Figure 2a,b). Therefore, the heat conductivity of the flexible thin tube is extremely high, which ensured the feasibility of ultrafast PCR thermal cycling.

Then, we tested whether it was possible to successfully amplify templates with the ultrafast thermal cycling process. As shown in Figure 2c, prior to the utPCR, no fluorescence was observed in any of the droplets. When the utPCR was performed with a 3 s cycle duration, some droplets exhibited fluorescence, but it was challenging to distinguish positive droplets from negative ones. However, when the cycle duration was extended to 5 s, droplets containing the DNA target displayed bright fluorescence, while the remaining droplets showed no fluorescence. Increasing the cycle duration from 5 s to 60 s resulted in all positive droplets emitting green fluorescence, clearly distinguishable from the negative ones. However, beyond 5 s per cycle, there was no significant increase in the fluorescence intensity of the positive droplets, indicating that 5 s per cycle was sufficient for primer annealing and enzyme extension during thermal cycling. Importantly, no bright droplets were generated in the utPCR amplification with no template added as a control (NTCs) (Figure S1). This demonstrated that there was no background signal when no template was present in the ultrafast amplification processes. To minimize the overall reaction time, we selected the 5 s per cycle process for the subsequent utPCR assay.

### 3.3. Rare Target Detection from High-Background Interference

**Figure 3:**
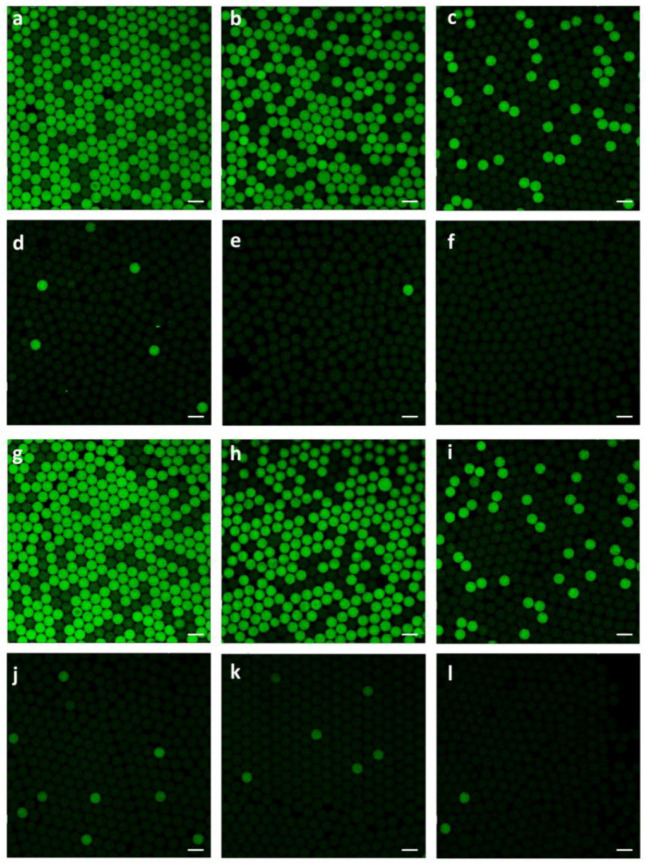
Comparison of utPCR (**a**–**f**) and standard droplet PCR (**g**–**l**) for *E. coli* O157 detection from high background of *E. coli* K12. From **a**–**e** (**g**–**k**), the ratio of *E. coli* to *E. coli* K12 were 1:10^2^, 1:10^3^, 1:10^4^, 1:10^5^, and 1:10^6^, with only *E. coli* K12 added as the negative control (**f**,**l**).

We further extended the utPCR system to quantify pathogenic *E. coli* O157 in the presence of a high background of normal *E. coli* K12 cells. The *E. coli* K12 concentration was kept at 5 × 10^6^ CFU per mL, while the *E. coli* O157 density varied from 5 × 10^4^ to 5 CFU per mL, corresponding to a range of 1:10^2^ to 1:10^6^ *E. coli* O157 to *E. coli* K12 ratios, challenging the sensitivity of the utPCR method. The sample containing only *E. coli* K12 at a concentration of 5 × 10^6^ CFU/mL served as the negative control. After the utPCR amplification, the results were analyzed using a fluorescent confocal microscope, with standard droplet PCR amplification as the control. The results demonstrated that, with the decrease of target *E. coli* O157 in each sample, the number of bright droplets reduced gradually (Figure 3a–e,g–j). Most importantly, for utPCR amplification, there was no bright droplet in the negative control (Figure 3f), though the non-template interference was at a high level. However, for standard droplet PCR, there were some fluorescent droplets displayed in the negative control (Figure 3l), which would greatly affect the final judgment of limit-of-detection (LOD). We assumed that nonspecific amplification would happen when non-template DNA existed in large amounts. Also, the long annealing time would increase the mismatch of primers to generate nonspecific amplification. Thus, utPCR illustrated higher detection accuracy and specificity than standard droplet PCR, especially for rare sample detection from high background interference. Because there was no false-positive for utPCR amplification, the initial template in the sample could be quantified by the quantity of positive droplets. In contrast, second-round PCR or further DNA sequencing was essential for standard droplet PCR when detecting rare targets from high background interference. Therefore, we primarily concluded that, with the ultrafast annealing time, utPCR demonstrated an advantage for rare template identification from high background interference.

### 3.4. Detection of Blood Stream Infection with the Proposed utPCR Method

**Figure 4:**
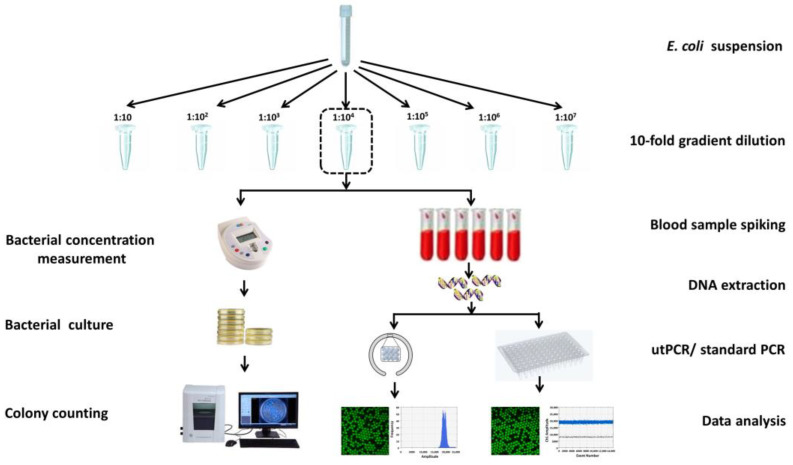
Workflow of blood sample spiked with *E. coli* O157 dilutions and pathogen detection with utPCR and standard droplet PCR amplification.

Advancing one step further, we challenged the system by detecting traces of *E. coli* O157 in blood samples. As is known, in early blood stream infection, it may carry as little as 1–100 pathogens against a high background of 10^7^ white blood cells per milliliter of blood. To simulate this condition, we spiked blood samples with 10-fold gradient diluted *E. coli* O157 from 10^6^ to 1 CFU per milliliter of blood, with no *E. coli* O157 spiked blood sample as the negative control (workflow can be seen in Figure 4).

**Figure 5:**
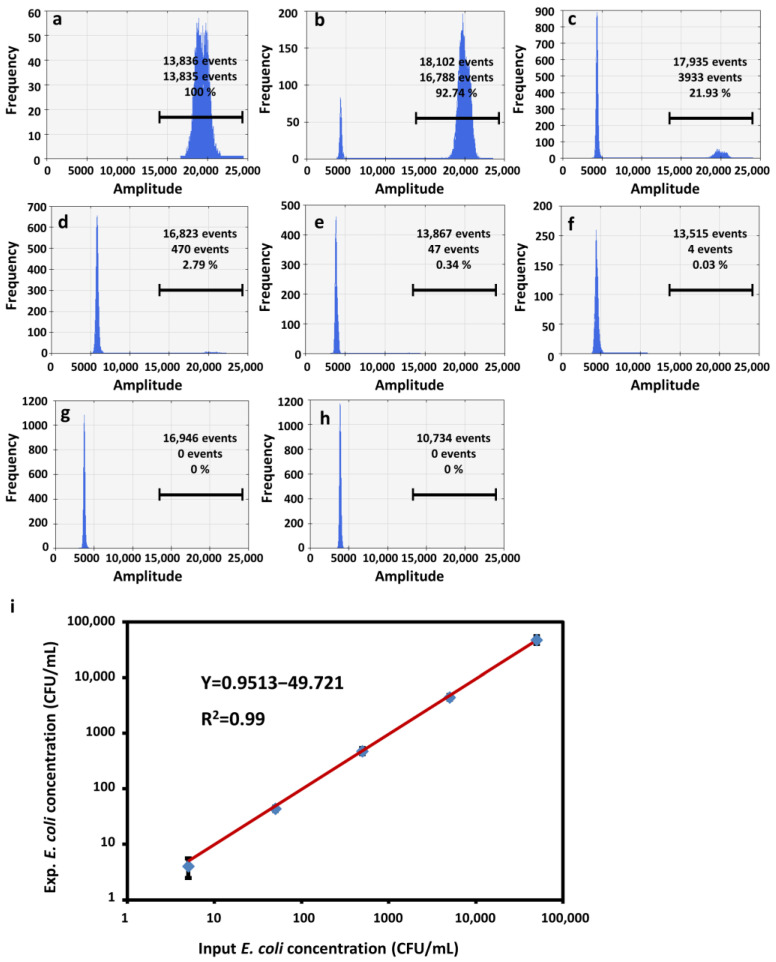
Fluorescence flow cytometry analysis of *E. coli* O157 in blood samples after utPCR amplification. The *E. coli* O157 concentration was gradually reduced by 10-fold from 10^6^ to 10^0^ CFU per milliliter of blood (**a**–**g**) with unspiked blood samples serving as the control (**h**). (**i**) Plot of the target pathogen concentration versus the measured pathogen concentration for *E. coli* O157 in blood samples using utPCR detection.

As expected, for utPCR the number of positive droplets was 10-fold decreased with the reduction of the *E. coli* O157 rate in each sample (Figure 5a–g and Figure S2). Most importantly, there were no positive events for the unspiked blood samples as the negative control (Figure 5h). Furthermore, we analyzed the number of *E. coli* O157 and compared the value with a theoretically added concentration in each sample. The results in Figure 5i display the background corrected calibration of *E. coli* O157 detection. The results showed a linear relationship between the input and measured *E. coli* O157 concentration ranging from 10 CFU/mL to 10^5^ CFU/mL with 99% confidence. The standard droplet PCR amplification process also displayed a linear relationship between theoretical and practical *E. coli* O157 concentrations when the spiked concentration was within the range of 10^5^–10^2^ CFU/mL (Figures S3 and S4). When the concentration of *E. coli* O157 was reduced to less than 100 CFU/mL, the detection result demonstrated a higher value than the theoretically spiked concentration. Moreover, for unspiked blood samples, there was also bright fluorescence existing in droplets. We assume that for standard droplet PCR amplification, because of the long annealing time, in addition to the high concentration of white cells as background, the standard reaction process may amplify nonspecific DNA that does not match the desired sequence. Therefore, utPCR demonstrated non-background, high amplification efficiency and detection accuracy, and the LOD could be as low as 10 CFU per milliliter of blood. Moreover, the large dynamic range of five magnitude orders should make utPCR a powerful tool for the accurate analysis of rare pathogens from high backgrounds.

## 4. Discussion

In this study, we have developed a novel strategy that can be completed in less than 5 min. This approach utilizes the high surface-to-volume ratio of droplets, enabling rapid temperature elevation and reduction. Additionally, we have designed a rapid thermocycling strategy by shuttling the thin tube containing droplets between low and high-temperature zones at high speed to achieve amplification. During ultrafast thermocycling, the primer annealing time has been significantly reduced from minutes to seconds, effectively eliminating potential nonspecific amplification induced by primers. Theoretically, all droplets in negative samples will remain nonfluorescent, allowing for single-molecule detection. Furthermore, by dispersing individual targets into millions of droplets, the background interference, such as DNA from white cells, can be simultaneously distributed among the droplets, thereby relatively reducing background interferences in ultrafast ddPCR. Based on these advancements, our proposed system enables single-molecule detection and absolute quantification. It is ultrafast, highly sensitive, and capable of accurately quantifying trace samples in the presence of high background interference, making it highly promising for biomedical applications.

As we all know, PCR is considered as the gold standard and plays a significant role in molecular diagnostics. Over the past four decades, it has evolved from end-point PCR to real-time PCR, and ultimately to absolute quantitative digital PCR [27,28]. To enable ultrafast and single-molecule detection, our focus centered on three crucial steps of digital PCR: sample dispersion, amplification, and quantification.

In terms of sample dispersion, the droplet-based method offers distinct advantages compared to microwell-based, channel-based, and printing-based methods. It allows for easy control of droplet size and composition, ensuring uniformity and rapid dispersion. In our study, we successfully generated monodisperse droplets with four different diameters, with most of the droplet generation process completed within 5 min for 25 μL reaction volume. Precise control of the droplet diameter was achieved by adjusting channel size and liquid flow rate. Additionally, the stability of droplets was greatly influenced by the surfactant ratio. In our assay, we utilized a 5% surfactant concentration, resulting in uniformly stable droplets after PCR thermocycling. Similar findings have been reported in the literature, stating that a surfactant ratio of no lower than 5% effectively maintains monodisperse droplet uniformity during heating incubation [29,30]. Moreover, the addition of 5% surfactant would not influence PCR amplification (Figure S5).

Amplification and quantification in digital PCR typically require several hours, which significantly hampers its application, particularly for rapid detection purposes. Additionally, the presence of unavoidable background signals in negative controls poses a significant challenge, particularly in single-molecule detection scenarios. Our preliminary results demonstrated that rapid PCR amplification exhibited no detectable background, unlike the standard amplification process [10]. We hypothesize that this is attributed to the prolonged annealing and extension times employed in the standard digital PCR amplification protocol. To address these limitations, we leveraged the high specific surface area of microscaled droplet systems and facilitated ultrafast heat transfer, resulting in a remarkable reduction in digital PCR amplification time from hours to only minutes. Crucially, by shortening the primer annealing and extension time from minutes to seconds, we achieved the complete absence of fluorescence signals in samples without the target. This significantly enhanced the differentiation of positive droplets and rendered our method highly suitable for single-molecule detection applications.

In future studies, it will be essential to address the potential issue of liquid barrier disruption among aqueous droplets during transfer and thermal cycling processes. To achieve this, we aim to enhance the sophistication and multifunctionality of our approach, particularly in terms of device design, optimization, fabrication, and system integration.

## 5. Conclusions

In conclusion, we have developed a novel utPCR strategy that enables the highly sensitive, specific, and ultrafast detection of single molecules in the presence of high background interference. By capitalizing on the advantages of ultrafast annealing/extension, this streamlined method demonstrates remarkably high amplification specificity. As a result, it enables the sensitive detection and digital quantification of *E. coli* O157, even in the presence of a 10^6^-fold excess of *E. coli* K12 cells. Furthermore, the method exhibits promising potential for the detection of rare pathogens in blood samples, achieving a remarkable limit of detection as low as 10 CFU/mL of blood with no background signals detected in negative controls. In addition, the proposed method also facilitates ultrafast and high-throughput droplet generation and analysis, which holds significant application potential in various areas such as the detection of rare pathogens in blood samples, elucidating drug-resistant mechanisms for drug development, and enumerating rare circulating tumor cells for early diagnosis. The versatility and capabilities of this method make it a promising tool for the next generation of molecular diagnosis.

## Figures and Tables

**Table 1:** The parameters of the device and relative flow rates to generate monodisperse droplets with different diameters.

Device	Oil Phase Width (μm)	Water Phase Width (μm)	Channel Depth (μm)	Oil Phase Speed (μL/h)	Water Phase Speed (μL/h)	Droplet Diameter (μm)	Total Reaction Volume (μL)	Total Time (min)
1	50	100	50	800	400	120	25	3.75
2	50	100	25	1200	400	80	25	3.75
3	15	30	25	800	400	37	25	3.75
4	8	8	8	200	100	15	25	15

## Data Availability

All data are contained within the article or the Supplementary Materials.

## Supplementary Materials

The following supporting information can be downloaded at: https://www.mdpi.com/article/10.3390/bios13100910/s1, Figure S1: Confocal images of negative samples after utPCR amplification; Figure S2: Fluorescence flow cytometry analysis of *E. coli* O157 in blood samples after utPCR amplification. Figure S3: Fluorescence flow cytometry analysis of *E. coli* O157 in blood samples after standard droplet PCR amplification. Figure S4: Plot of target pathogen concentration vs. measured pathogen concentration for *E. coli* O157 detection by standard droplet PCR amplification; Figure S5: The influence of surfactant as additive for real-time PCR amplification; Table S1: Reaction conditions of utPCR and standard droplet PCR amplification.
