# Supplementary material for: utPCR: A Strategy for the Highly Specific and Absolutely Quantitative Detection of Single Molecules within Only Minutes
Source: Biosensors (Basel). 2023 Sep 27;13(10):910. doi: 10.3390/bios13100910 (PMC10605045; doi:10.3390/bios13100910)
Supplement: Supplementary file 1 [file biosensors-13-00910-s001.zip › Biosensors ESI Revised.pdf]

Electronic Supplementary Information for

utPCR: A Strategy for the Highly Specific and Absolutely Quantitative Detection of Single Molecules within Only Minutes

Rui Wang [1,2], Ying Liu [1], Shuaiwei Chen [1], Linlin Bai [1], Kaiming Guo [1], Yanan Pang [3], Feng Qian [1], Yongfang Li [4], Li Ding [3,*] and Yongming Wang [1,5,*]

[1] State Key Laboratory of Genetic Engineering, School of Life Sciences, Zhongshan Hospital, Human Phenome Institute, Pudong Hospital, Fudan University, Shanghai 200438, China; wangr@fudan.edu.cn (R.W.); liuyinglyac@163.com (Y.L.); 21210700106@m.fudan.edu.cn (S.C.); 18437953528@163.com (L.B.); 18307110089@fudan.edu.cn (K.G.); fengqian@fudan.edu.cn (F.Q.)

[2] School of Engineering and Applied Sciences, Harvard University, Cambridge, MA 02138, USA

[3] Changhai Hospital, Second Military Medical University, Shanghai 200433, China; 15721259472@163.com

[4] School of Food Science and Engineering, Foshan University, Foshan 528231, China; 18110220111@fudan.edu.cn

[5] Shanghai Engineering Research Center of Industrial Microorganisms, Shanghai 200438, China

* Correspondence: xuanyu_1119@sina.com (L.D.); ymw@fudan.edu.cn (Y.W.)

Figure S1 Confocal images of negative samples after utPCR amplification.

Table S1 Reaction conditions of utPCR and standard droplet PCR amplification.

Figure S2 Fluorescence flow cytometry analysis of *E. coli O157* in blood samples after utPCR amplification.

Figure S3 Fluorescence flow cytometry analysis of *E. coli O157* in blood samples after standard droplet PCR amplification.

Figure S4 Plot of target pathogen concentration *vs.* measured pathogen concentration for *E. coli O157* detection by standard droplet PCR amplification.

Figure S5 The influence of surfactant as additive for real-time PCR amplification.

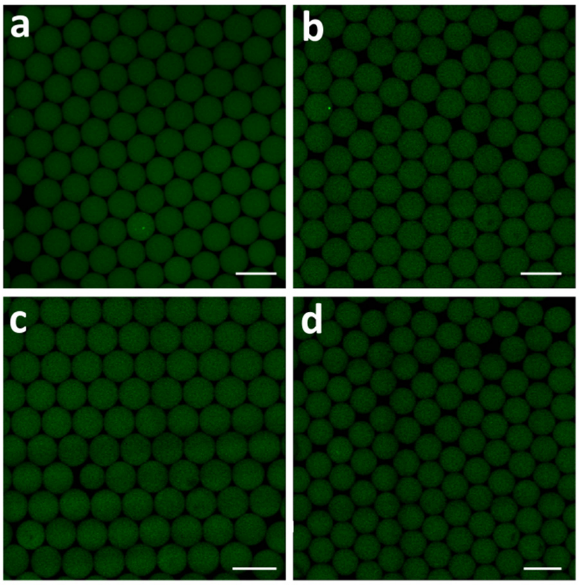

Figure S1 Confocal images of negative samples (no template added) after utPCR amplification. From a-d, utPCR was carried out with 3s per cycle, 5s per cycle, 10 s per cycle and 60 s per cycle, respectively.

Table S1 Reaction conditions of utPCR and standard droplet PCR amplification.

Reagent	Work concentration	
	Droplet rapid-PCR	Droplet standard-PCR
DNA polymerase*	10 U	1 U
Tris HCl (pH 8.3)	10 mM	10 mM
KCl	50 mM	50 mM
$MgCl_2$	1.5 mM	1.5 mM
dNTP (each)	0.2 mM	0.2 mM
Forward primer	0.4 μM	0.4 μM
Reverse primer	0.4 μM	0.4 μM
Eva Green	1×	1×
10 % Tween 20	0.5 μL	0.5 μL
BSA	0.5 μL	0.5 μL
Template	10 μL	10 μL
Sterile water	Up to 25 μL	Up to 25 μL

*The concentration of DNA polymerase in utPCR was ten times that of standard PCR.

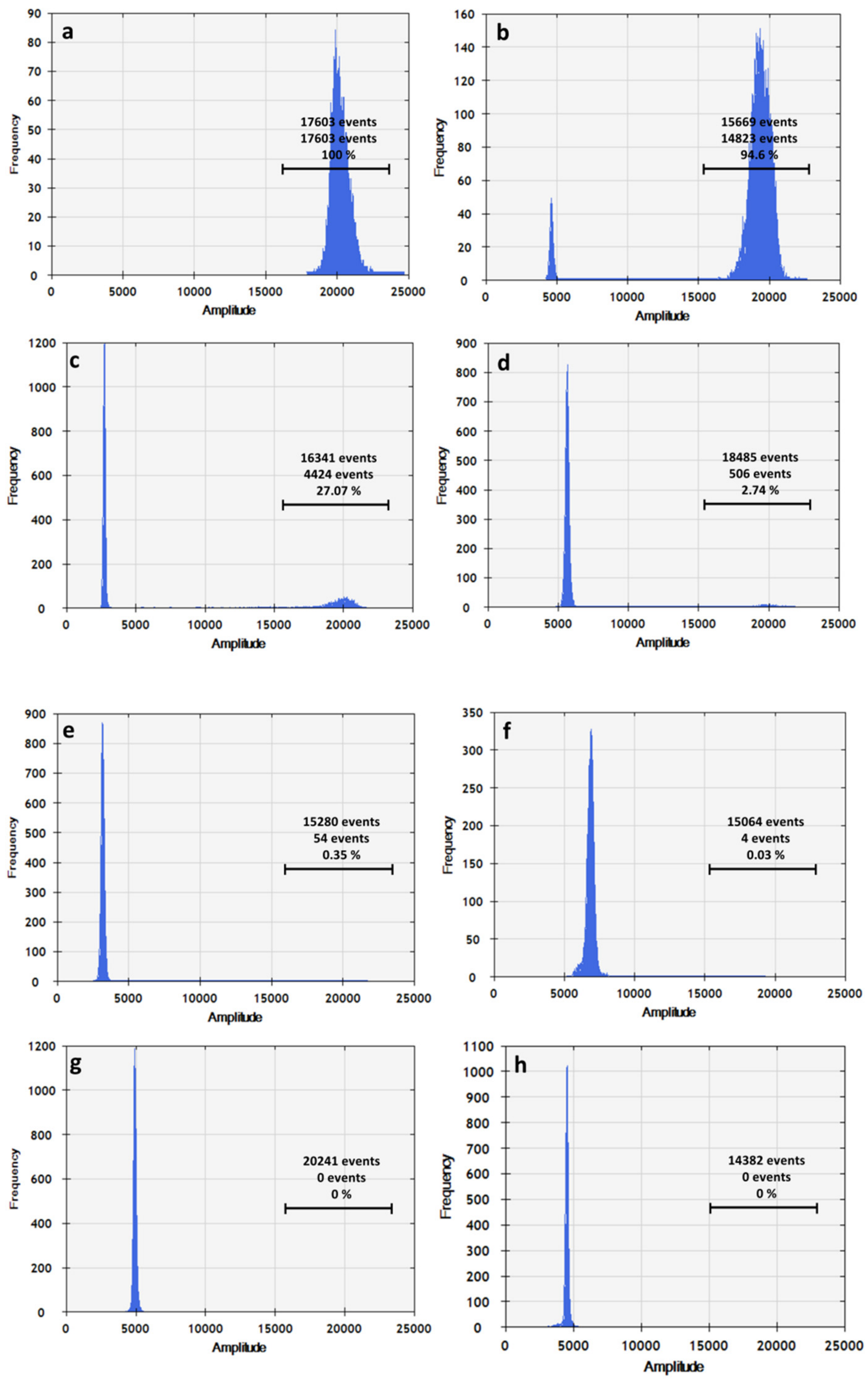

Figure S2 Fluorescence flow cytometry analysis of *E. coli O157* in blood samples after utPCR amplification. The same letter as that in **Figure 5** (for example, the letter “a”) represents different aliquots from the same blood sample. The concentration of *E. coli O157* was 10-fold gradually reduced from 10^6 to 10^0 CFU per mL blood (a-g) with unspiked blood sample as control (h).

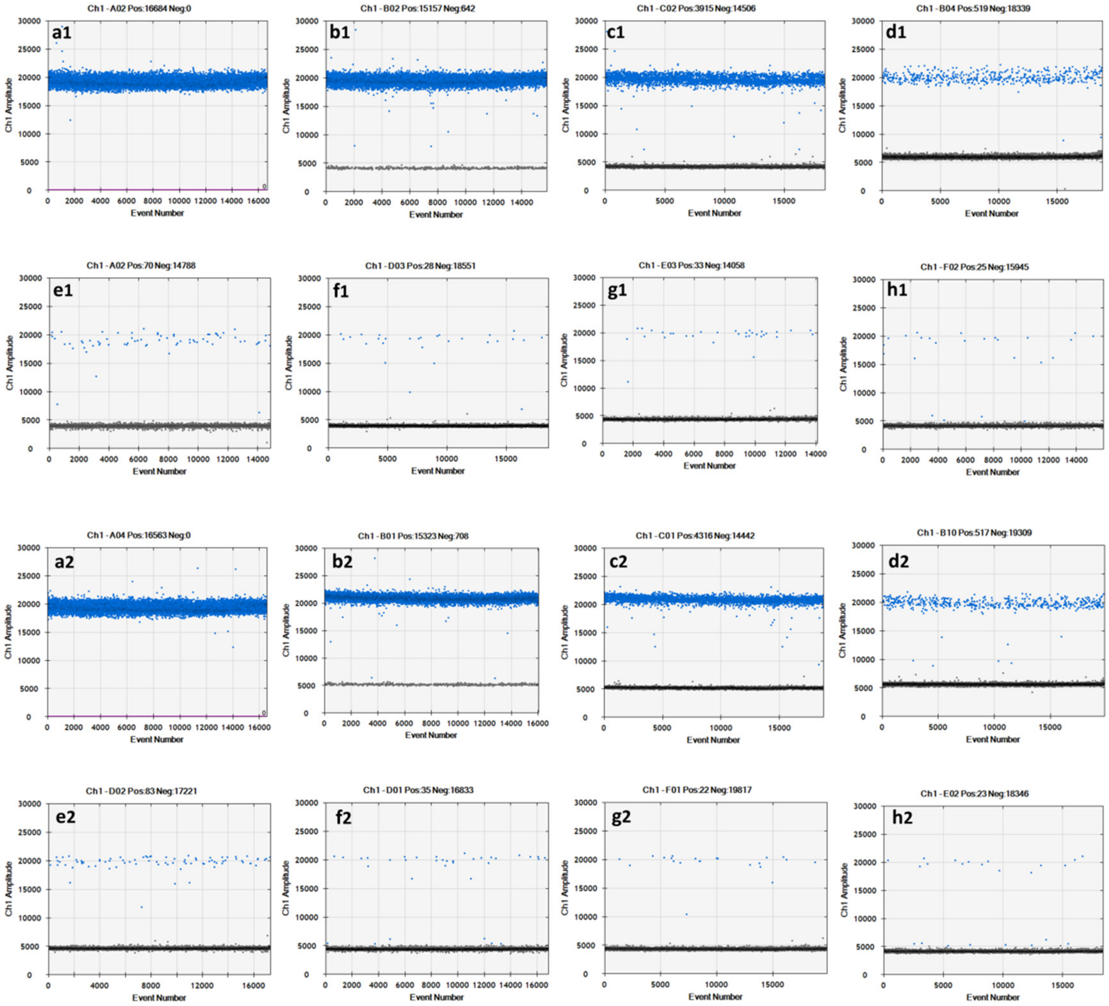

Figure S3 Fluorescence flow cytometry analysis of *E. coli O157* in blood samples after standard droplet PCR amplification. The same letter (for example a1 and a2) represents different aliquots of the same blood sample. The concentration of *E. coli O157* was 10-fold gradually reduced from 10^6 to 100 CFU per mL blood (a-g) with unspiked blood sample as control (h).

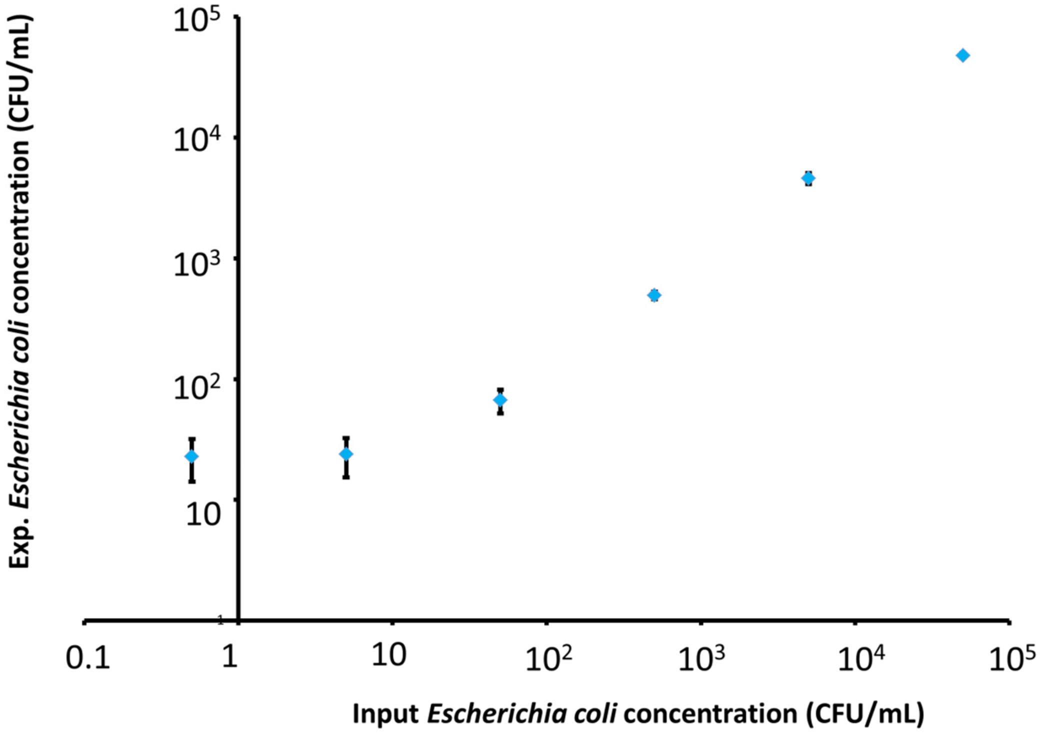

Figure S4 Plot of target pathogen concentration *vs.* measured pathogen concentration for *E. coli O157* detection by standard droplet PCR amplification.

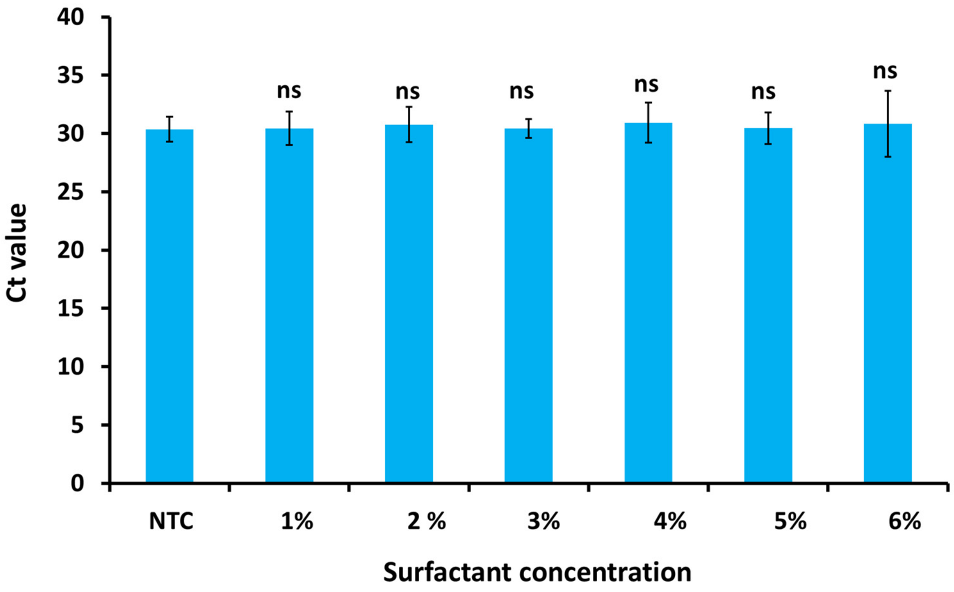

Figure S5 The influence of surfactant as additive for real-time PCR amplification with the same concentration of template. NTC, no surfactant added as negative control. n=3, error bars showed mean± SEM).
